# Natal and breeding philopatry of female Steller sea lions in southeastern Alaska

**DOI:** 10.1371/journal.pone.0176840

**Published:** 2017-06-07

**Authors:** Kelly K. Hastings, Lauri A. Jemison, Grey W. Pendleton, Kimberly L. Raum-Suryan, Kenneth W. Pitcher

**Affiliations:** 1 Alaska Department of Fish and Game, Division of Wildlife Conservation, Anchorage, Alaska, United States of America; 2 National Marine Fisheries Service, Protected Resources Division, Juneau, Alaska, United States of America; University of Illinois at Urbana-Champaign, UNITED STATES

## Abstract

Information on drivers of dispersal is critical for wildlife conservation but is rare for long-lived marine mammal species with large geographic ranges. We fit multi-state mark-recapture models to resighting data of 369 known-aged Steller sea lion (*Eumetopias jubatus*) females marked as pups on their natal rookeries in southeastern Alaska from 1994–2005 and monitored from 2001–15. We estimated probabilities of females being first observed parous at their natal site (natal philopatry), and of not moving breeding sites among years (breeding philopatry) at large (> 400 km, all five rookeries in southeastern Alaska) and small (< 4 km, all islands within the largest rookery, Forrester Island Complex, F) spatial scales. At the rookery scale, natal philopatry was moderately high (0.776–0.859) for most rookeries and breeding philopatry was nearly 1, with < 3% of females switching breeding rookeries between years. At more populous islands at F, natal philopatry was 0.500–0.684 versus 0.295–0.437 at less populous islands, and breeding philopatry was 0.919–0.926 versus 0.604–0.858. At both spatial scales, the probability of pupping at a non-natal site increased with population size of, and declined with distance from, the destination site. Natal philopatry of < 1 would increase gene flow, improve population resilience, and promote population recovery after decline in a heterogeneous environment. Very high breeding philopatry suggests that familiarity with neighboring females and knowledge of the breeding site (the topography of pupping sites and nearby foraging locations) may be a critical component to reproductive strategies of sea lions.

## Introduction

The process of dispersal has long been recognized as a critical component to population viability by allowing individuals to move in response to environmental conditions that are heterogeneous in time and space [1–3], or to avoid detrimental consequences to population viability and individual fitness, such as inbreeding or kin competition (reviewed by [4]). More recently, understanding dispersal ability has been recognized as critical for anticipating potential range shifts and other population responses resulting from current climate change [5–6]. Although marine birds and mammals are highly vagile and their dispersal patterns are largely determined by individual behavior rather than physical barriers, movements of colonially-breeding species are restricted at least seasonally by their need for physical grouping with conspecifics [7], often coupled with polygynous mating systems in the case of marine mammals [8]. Colonies also often result from the requirement of productive foraging grounds near breeding sites [6], and this is especially true for otariids (sea lions and fur seals) in which females must feed while lactating [8].

Like most mammals, marine mammal populations are generally characterized by male-mediated gene flow via male dispersal [4, 9]. However, dispersal patterns of females may also affect population structure [10–12] and status, particularly by slowing population growth and restricting breeding distribution [7]. Generally, fidelity to the natal site (giving birth at their natal site) or breeding site (repeatedly giving birth at the same site) is high for females of colonially-breeding marine mammals [13], with some demonstrating fine-scale philopatry to breeding locations that may vary annually by only meters [14–17]. Although studies of genetic population structure are useful for examining dispersal patterns [12], the structure results from multiple processes including historical processes, genetic drift, and mutation, as well as current patterns of dispersal [18]. In addition, genetic patterns might change over a longer time scale than behavioral patterns. Describing current dispersal patterns through direct estimates of movement probabilities based on long-term study of individuals is rare in pinnipeds, particularly covering multiple breeding sites for long-lived species with large breeding ranges. For these species, accurate estimates accounting for survival and spatial variation in resighting probabilities often require mark-recapture studies of individually-marked animals [19].

Steller sea lions (SSL, *Eumetopias jubatus*) breed in a near linear series of rookeries along the coast of the North Pacific from California, around the Gulf of Alaska and Aleutian Islands, to the Okhotsk Sea and Kuril Islands, Russia (n = ~50 rookeries, [20–21]). Population structure at the rookery (geographically distinct breeding aggregations, <50 km), region (regional clusters of rookeries, >100 km), and stock (large-scale management units, >> 200 km) levels has been demonstrated by phylogenetic studies [21–24]. Genetic and demographic data suggest three stocks of SSL (Russian, western, and eastern, [21, 25–26]) marked by higher dispersal rates for males than females [26–28] and moderate to high female philopatry over limited spatial scales [21, 26]. The size of the western and Russian stocks (north and west of southeastern Alaska, SEAK) declined dramatically from the mid-1970s to 2000 and the western stock is now recovering or stabilized in much of its range [29–31]. The eastern stock (SEAK through California) has grown moderately during the same period, including the stabilization of numbers at the largest and previously northernmost rookery, Forrester Island Complex (F, ~4000 pups produced annually [31]), and the appearance of four new breeding rookeries to the north of F [32]. The two northernmost of these new rookeries were formed by immigration of breeding females from both the eastern and western stocks [28, 33].

Although moderate to high levels of natal and breeding philopatry for female SSL can be inferred from genetic and population data, few estimates exist of current rates of movement for pupping females. Return rates of a small sample from Marmot Island (western stock) and F (*n* = 12 and 29, respectively) suggested only moderately high female natal philopatry, with 67% and 81% of females, respectively, first observed parous at their natal rookery versus other monitored rookeries [26]. Here, we analyzed mark-recapture data for 369 known-aged females marked as pups on their natal rookeries in SEAK from 1994–2005 and monitored from 2001–15 to estimate probabilities of females being first observed parous at their natal rookery (natal philopatry), and probabilities of females not moving pupping rookeries between years (breeding philopatry) for all five rookeries in SEAK, ranging up to > 400 km apart. We also examined natal and breeding philopatry at a finer spatial scale (< 4 km) among the five islands at F at which females pup.

## Methods

**Fig 1:**
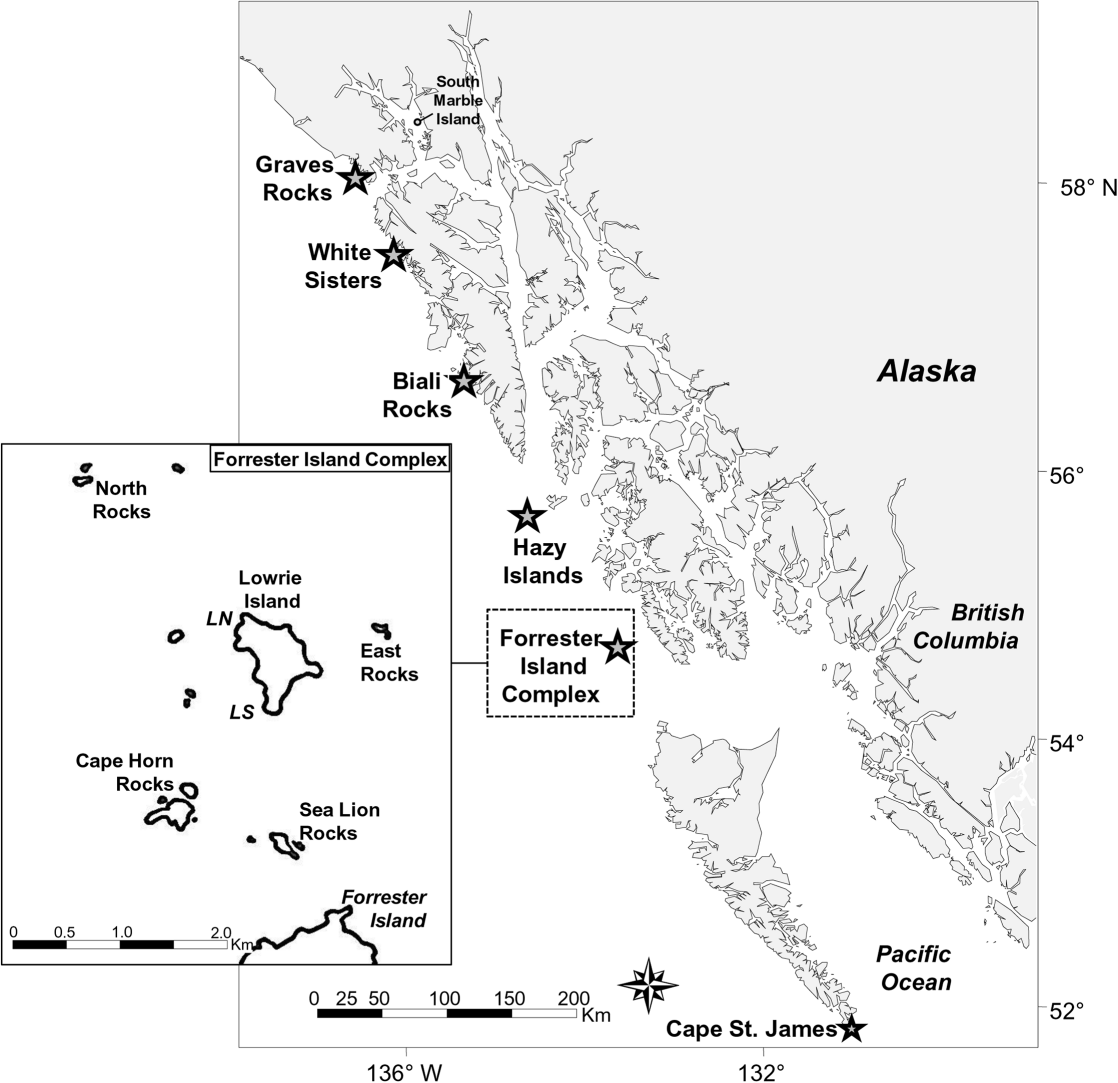
Map of Steller sea lion rookeries in southeastern Alaska, and islands at which female Steller sea lions pup at the Forrester Island Complex rookery. Rookeries are shown with a star (Forrester Island Complex = F, Hazy Islands = H, Biali Rocks = B, White Sisters = W, Graves Rocks = G). The Cape St. James rookery in northern British Columbia is also shown. The inset shows the five island groups where females pup at the Forrester Island Complex rookery. Two pupping areas on Lowrie Island were LN (Lowrie Island North) and LS (Lowrie Island South).

In Alaska, SSL females give birth from late May–early July and pupping is highly synchronous. For example, nearly 70% of births occurred between 5 and 16 June on two islands in the Gulf of Alaska [34]. To provide a known-aged sample for population studies, female pups were branded in late June–early July (2–4 weeks of age) at four of five rookeries in SEAK (F = Forrester Island Complex, H = Hazy Islands, W = White Sisters, G = Graves Rock, Fig 1) in 1994–95 (*n* = 372) and 2001–05 (*n* = 890). No pups were branded at Biali Rocks (B), the smallest and newest rookery (Fig 1). Pup counts in 2015 at these five rookeries were: F—3954 pups (rookery established = ~first year > 30 pups counted < 1961, before systematic monitoring began), H—1994 pups (est. ~1979), W—910 pups (est. ~1990), G—502 pups (est. ~2002), and B—204 pups (est. ~2002) [31–32].

We observed branded females in SEAK from May–July from 2001–15 during large scale boat-based surveys covering all rookeries and haul-outs (terrestrial sites where SSLs routinely come ashore to rest, but where few or no pups are born) [28, 35–36]. Resighting effort was especially high at F, where an annual field camp from May–July at Lowrie Island (LI) provided near-daily surveys of LI, and in 2005 and 2007–14 weekly boat-based surveys of the four additional islands where females pup at F (Fig 1). Boat-based surveys at F were few in 2015 and occurred only after 1 July. Rookeries other than F were surveyed over one to two days per year before 2005. From 2005–15, three to six reproductive surveys/rookery/year were conducted in July after pupping was completed, to increase probability of sighting pups with females and allow reproductive rate estimates. These surveys equalized effort (reduced heterogeneity) among females observed and explicitly documented effort per female. The quality of pup sightings was also documented by explicitly recording specific behaviors. SSL females often form a distinct pair with their pup on the crowded rookery, especially when the pup is very young. However, after the perinatal period (the period following parturition when females remain ashore with their pup, mean length of 7.9 days at LI in 1994–95 [37]), females leave rookeries daily to forage, leaving their pups unattended on the rookery. Pups and mothers also are regularly physically separate from each other even when both are present on the rookery, and females may also lie next to or interact with pups other than their own, especially as the pup ages.

Every time a female was judged to have a pup, the best behavior (most definitive that the female had a pup) observed was recorded. Best behavior was: birth > nursing > pup lying on the back of the female > reunion of mother and pup, and/or movement together around the rookery > repeated or extensive behavioral interaction (nuzzling, sniffing) > brief interaction > pup lying next to female in an obvious pair > female alone or status unknown (especially due to poor view). By determining female-pup association by these behaviors, we assumed fostering of non-related pups by females was negligible. Fostering has only rarely been documented in SSL [34, 38] and female SSL are commonly aggressive towards pups not their own [39]. Branded females were photographed on every observation and their identities were confirmed with a photograph library, as misshapen digits may occur when brands heal creating identification error [36]. Animal observation and marking procedures were reviewed and approved under U.S. Marine Mammal permits issued to the Alaska Department of Fish and Game.

We used multi-state mark-recapture models [19] and treated site as a state variable to estimate probabilities of apparent survival (*S*), resighting (*p*), and moving between sites (*ψ*, the parameter of interest). We included only sightings of females judged to be definitively with a pup in a given year (best behavior observed that year was ≥ extensive interaction), and therefore *p* was confounded with pup detection and birth probability. *S* was also confounded with reproductive senescence (i.e., females that stopped pupping would appear to be dead). Confounding between mortality and emigration from the study area was likely negligible as very few SEAK-born females used areas to the north or west of 144° longitude and have never been observed pupping there despite high survey effort [26, 28]. Survey effort was lower at British Columbia rookeries to the south of our study area, which were surveyed intermittently from 2000–13. Only two SEAK-born females were recorded with pup there: one seen twice only in her life, both times in British Columbia (including once with weak evidence of pup at Triangle Island ~530 km south of F), and one seen definitively with pup at Cape St. James (~365 km from F, Fig 1) at age six, then regularly with pup at F after age six.

We created annual encounter histories by summarizing multiple sightings of a female definitively with a pup in a year (average and maximum of 2 and 8 times per year) as a single annual sighting. In encounter histories, a female definitively with a pup in a given year was coded as a letter indicating the location where she was observed and a female not seen or not seen definitively with a pup in a year as 0. To address natal philopatry, we artificially created sightings at the natal location in the year prior to females' first year observed with pup, and these served as females' first release in the analyses. Therefore, movement between the natal location and the first observed pupping location (natal philopatry) was indicated by *ψ* for the first release interval and all females were necessarily survivors of the interval and seen on the next occasion (so we fixed *S* for the first interval and *p* of the next occasion to 1). After the first release interval, *ψ* for other intervals indicated movement between pupping locations, for females that had pupped at least once previously (breeding philopatry). We used Program MARK [40] and RMark [41–42] to fit models and estimate parameters, and selected models based on AICc Weight [43–44]. We examined goodness-of-fit of the most complex models fit to the two datasets using the median $\hat{c}$ procedure in program MARK [45].

### SEAK analysis

To examine SEAK-wide patterns, we created encounter histories of 15 years (2001–15) for each female ever seen definitively with pup in May–July from 2002–15 (*n* = 1,274 sightings of 370 females). Only seven females were definitively with pup in 2000–01 and so capture histories began in 2002, as *p* would not be estimable for those years. No branded females were observed moving with their pup among different rookeries within a breeding season before 01-Aug. Also, only one of 370 females (0.3%), W330, was seen with a pup at a haul-out (South Marble Island in Glacier Bay National Park, Fig 1) and in only one year. Similarly, very few pups were observed at haul-outs during count surveys (0.2%) [32]. Therefore we excluded all data of W330, and in encounter histories, coded a female with pup in a given year as a letter indicating the rookery she was at (F, H, B, W, or G). One female from F was observed extensively interacting with a pup at age three on only one occasion very late in the season (20 July). We discounted this observation as an error as we did not observe any other three year old nurse or give birth to a pup in SEAK, and we considered her next year definitively (nursing) with pup (at age 6) as her first pup.

Using a base *ψ* model that estimated all possible transitions for the two philopatry "types" (natal versus breeding philopatry) and the most complex *S* model, we first (step 1) fit 10 *p* models allowing *p* to vary among years, rookeries and with female age. Female age was structured five ways: constant across ages, <15 years versus 15+ years (two categories), a linear or quadratic trend in *p* with age, and a B-spline-fit of age to allow a complex pattern [46]. We then (step 2) fit 10 *S* models to the best *p* model, allowing *S* to vary among two rookery groups (F/H/B versus W/V, based on results in [36]) and with female age in the same manner as *p*.

Next (step 3), we fit an additional *ψ* model with the best *S* and *p* models to allow age effects in *ψ* in which all *ψ* were estimated separately for females aged 5–7 versus 8+ years. This model addressed our concern that the older cohort of females born in 1994–95 could only enter the study at an older age (10–11 years in 2005), whereas the younger cohort born in 2001–05 were surveyed at both younger and older ages (4–14 years), including their potential first years of reproduction. We wished to ensure that natal philopatry was not biased high by the entry of the older cohort into the study only at ≥10 years of age, as would occur if natal philopatry increased with female age or experience.

Finally (step 4), we fit additional *ψ* models based on the best model from step 3 to examine if probability of breeding at a non-natal rookery was a function of natal rookery, distance between the natal and non-natal rookeries (linear or quadratic effects), and pup population size (using pup counts from 2015 from [31]) of the destination rookery (linear effect). We used Google Earth to estimate the shortest straight-line distance between rookeries. We fit all possible combinations of non-interacting variables, assuming common slopes among natal rookeries and allowing common and varying intercepts among natal rookeries.

### F analysis

To examine patterns at F only, we created encounter histories of 11 years (2004–15, excluding 2006) for all females that were ever seen definitively with pup at F from May– 05-July in 2005 and 2007–15. This included 183 females originally-born at F from 1994–2004 (at islands: Lowrie Island: LI, *n* = 54; Cape Horn Rocks: CHR, *n* = 44; Sea Lion Rocks: SLR, *n* = 16; and North Rocks: NR, *n* = 62; Fig 1), and also few females not born at F: from H (*n* = 5) and W (*n* = 2). We used data from these years because surveys of all islands for the entire breeding season began in 2005, and data collection in 2006 was halted by legal injunction. We coded a female with pup in a given year as a letter indicating where she was first seen with a pup (six possible areas—LI north, LN; LI south, LS; CHR; SLR; NR; East Rocks, ER; Fig 1). We coded females from natal rookeries H or W (*n =* 7) as X in capture histories to remove these few born at other rookeries from natal philopatry estimates. Therefore, we mainly address island philopatry at F, but also divided one island, LI, into two "areas", north versus south (LN and LS), to be consistent with other studies [47]. No pups were marked at LS or ER.

Using the same base *ψ* model as in the SEAK analysis, and an *S* model including age effects (in the best structure observed for the SEAK data), we fit (step 1) four *p* models, which all included female age (in the best structure observed for the SEAK data) but allowed *p* of females with pups at LN/LS/NR/Islands1 (Islands1: CHR, SLR, ER) to be separate or pooled in various ways. We expected high *p* at LI versus islands surveyed by boat (Islands2: NR, CHR, SLR, ER), due to higher effort and the better observation platform. We expected lower *p* at LS than LN due to crowding and poorer vantage points at LS versus LN. We expected lower *p* at NR than other boat-surveyed islands (Islands1) due to low-lying geography and rough waters at NR. For all four *p* models, an additional variable was included to allow lower *p* for boat-surveyed islands in 2015 as effort was very low for these sites in this year. We then fit (step 2) the same additional *ψ* model as for the SEAK analysis, allowing age effects in *ψ*. As a post-hoc analysis, we used generalized linear models (binomial error distribution, logit link) to determine statistical support for whether females had a preference for first pupping at their natal island, as indicated by higher natal fidelity than expected due to the relative pup abundance at islands alone (e.g. if 20% of the total pups born at F were born at island *x*, and natal fidelity of pups born at *x* was 40%, a preference may be indicated even though the point value of 40% may appear low by value alone). The response variable in these models was the estimated number of females first observed with pups at each natal island *x* breeding island combination (20 data points, calculated as number released per natal island *x ψ*,). We fit a base model including the predictive covariate, pup population size in 2015 (two parameters). We then fit a model also including the variable "natal island", as a binary predictor variable (natal island versus non-natal island, three parameters). The three parameter model was preferred if ΔAICc was > 2.

Finally (step 3), as for the SEAK analyses, we fit additional *ψ* models based on the best model from step 2 to examine if probability of breeding at a non-natal/breeding island varied with natal/breeding island, distance between the natal and non-natal/breeding islands, and pup population size of the destination island.

## Results

The median $\hat{c}$ procedure estimated $\hat{c}$ = 1.25 and 0.99 for the most complex models fit for the SEAK and F analysis, respectively. To run these models, rookery or site codes in encounter histories were recoded as 1 and the models were fit as Cormack-Jolly-Seber models (no state variable and only parameters *S* and *p* estimated) and therefore did not include rookery or site effects. Although mild heterogeneity is suggested for the SEAK analysis, important site effects were ignored by the test and we suspected these effects largely contributed to the $\hat{c}$ > 1.0. Also, model selection results for the SEAK analysis were identical irrespective of the use of the 1.25 variance inflation factor.

### SEAK analysis

For the 369 marked females ever seen definitively with pup, sample sizes of females seen with pup per age were 9 (age 4), 82 (age 5), 104–163 (ages 6–12), 81 (age 13), 59 (age 14), 22–30 (ages 15–18), and 9–11 (ages 19–20). Sample sizes of those by natal rookery were 220 (F), 67 (H), 60 (W), and 22 (G). On average, females were seen definitively with pup in 3.5 years, and sample sizes of these females seen *x* number of years were 70–73 (1–3 years), 59 (4 years), 27–33 (5–6 years), 13–16 (7–8 years), and 1–4 (9–11 years).

The best models for nuisance variables *p* and *S* included rookery and quadratic age effects for *p*, and only a linear age effect for *S* (Table 1A and 1B). Rookery-specific estimates of *p* from model 15 (Table 1B) indicated that *p* of females with pups at B could not be estimated separately (95% confidence interval, CI, ranged 0–1), most likely due to very small sample size (*n* = 7 sightings of females with pups after the first initial pup sightings versus 613/119/93/72 at F/H/W/G). Post-hoc analyses determined that B should be pooled with H (with the lowest *p*) rather than F (with the highest *p*, Table 1C, model 21). *P* averaged 0.36, 0.15, 0.20, and 0.28 for females with pups at F, H/B, W, and G, respectively, and ranged up to ± 0.16 from the average among years. In 2014 for females with a pup at F, p* (female resighting rate confounded with pup detection and birth probability) was 0.47 at age 5, increasing to 0.76 at 12–13 and then decreasing to 0.47 at age 20. S* (survival confounded with breeding senescence) decreased -0.21 from age 4 to 19, and higher survival of W/G than F/H pupping females (see [36]) was not supported (Table 1B) as point estimates differed by ≤ 0.003 between the two rookery groups.

**Table 1:** Model selection results for estimating movement (ψ), survival (S) and resighting (p) probabilities for Steller sea lion females with pups at southeastern Alaska rookeries (SEAK, 2002–2015), and at Forrester Island Complex (F, 2005 and 2007–15).

Model#	Model	nPar	AICc	ΔAICc	AICc Weight
(A) SEAK: Modeling p					
with S (rookery2 + spline curve Age), ψ (rookery:torookery:np + rookery:torookery:bp)					
2	p (year + rookery + Age + Age2) *BEST	59	3571.89	0.00	0.66
1	p (year + rookery + spline curve Age)	60	3573.29	1.40	0.33
3	p (year + rookery + Age)	58	3580.36	8.47	0.01
5	p (year + rookery)	57	3607.74	35.85	0.00
(B) SEAK: Modeling S					
with p (BEST), ψ (rookery:torookery:np + rookery:torookery:bp)					
15	S (Age)	56	3567.30	0.00	0.44
16	S (Age + Age2)	57	3569.43	2.14	0.15
12	S (rookery2 + Age)	57	3569.45	2.15	0.15
17	S (spline curve Age)	58	3569.73	2.43	0.13
(C) SEAK: Modeling S, checking pooling of B with F or H					
21	S (Age) p (year + Age + Age2 + rookery (HB pooled)) *BEST	55	3565.14	0.00	0.52
15	S (Age) p (year + Age + Age2 + rookery (B not pooled))	56	3567.30	2.15	0.18
20	S (Age) p (year + Age + Age2 + rookery (FB pooled))	55	3568.77	3.63	0.08
16	S (Age + Age2) p (year + Age2 + rookery (B not pooled))	57	3569.43	4.29	0.06
(D) SEAK: Modeling effect of distance and population size on ψ					
with p (BEST), S (BEST)					
37	ψ (FHW:np + FHW:np:Distance + FHW:np:Distance2 + FHW:np:Popsize + G:np + rookery:bp)	29	3537.82	0.00	0.34
39	ψ (FHW:np + FHW:np:Distance + FHW:np:Popsize + G:np + rookery:bp) *BEST	28	3537.93	0.11	0.32
41	ψ (FHW:np + FHW:np:Distance + G:np + rookery:bp)	27	3540.88	3.06	0.07
38	ψ (FHW:np + FHW:np:Distance + FHW:np:Distance2 + G:np + rookery:bp)	28	3541.78	3.96	0.05
(E) F: Modeling p, pooling of sites					
with S (Age), ψ (island:toIsland:np + island:toIsland:bp)					
F1	p (year + Age + Age2 + LN/LS/NR/Islands1 + Isl2015)	72	2837.42	0.00	0.46
F2	p (year + Age + Age2 + LI/NR/Islands1 + Isl2015)	71	2837.63	0.21	0.41
F3	p (year + Age + Age2 + LI/Islands2 + Isl2015)	70	2841.18	3.77	0.07
F4	p (year + Age + Age2 + LN/LS/Islands2 + Isl2015)	71	2841.33	3.91	0.06
(F) F: Modeling effect of distance and population size on ψ					
with S (Age), p (year + Age + Age2 + LI/Islands2 + Isl2015)					
F9	ψ (island:np + island:bp + Distance + Popsize)	32	2496.13	0.00	0.93
F11	ψ (island:np + island:bp + Distance)	31	2501.27	5.14	0.07
F12	ψ (np + island:bp + Distance + Popsize)	29	2509.09	12.96	0.01
F6	ψ (island:np + island:toIsland:bp + np:Distance + np:Popsize)	47	2511.42	15.29	0.00

nPar = number of parameters in the model, AICc = Akaike's Information Criterion corrected for small sample size, ΔAIC = difference in AICc from the top model, AICc Weight = AICc weight, compared to other models. Only top 4 models are shown. np = natal philopatry (first observation parous), bp = breeding philopatry (subsequent observations parous), Age = linear fit of Age, Age2 = quadratic fit of Age, spline curve Age = B-spline fit of Age, rookery2 = F/H/B versus W/G, H = Hazy Islands, F = Forrester Island Complex, B = Biali Rocks, W = White Sisters, G = Graves Rocks, LN = Lowrie Island North, LS = Lowrie Island South, LI = Lowrie Island (LN and LS pooled), NR = North Rocks, Islands1 = Cape Horn Rocks, Sea Lion Rocks and East Rocks, Islands2 = those in Islands1 + North Rocks, Isl2015 = seen at Islands2 in 2015.

Natal philopatry for SEAK rookeries was highest at G (1.000), which had the smallest sample size (*n* = 22 females), next highest at F (0.859, *n* = 220) and lowest at H and W (~0.780, *n* = 67 and 60, Table 2). In contrast, breeding philopatry was nearly 1 for all major rookeries with < 0.03 changing pupping sites between years (Table 2). In contrast, females with pups at B were more likely to switch rookeries than those with pups at other sites (0.14 switching), although sample size of females with pups at B was very small (*n* = 16 sightings). The inclusion of the age effect in *ψ* was not supported (model 21, Table 1C = ψ (rookery:torookery:np + rookery:torookery:bp): AICc = 3565.14 versus same model with age effect, not shown in Table 1 = ψ (rookery:torookery:np:age + rookery:torookery:bp:age): AICc = 3612.09).

**Table 2:** Estimates of natal and breeding philopatry for female Steller sea lions in southeastern Alaska, 2002–15.

	*To*: Breeding rookery (South → North)				
*From*:* *	F	H	B	W	G
Natal philopatry					
Natal rookery:					
F	0.859	0.105	0.009	0.018	0.009
	(0.807–0.899)	(0.070–0.152)	(0.002–0.036)	(0.007–0.047)	(0.002–0.036)
H	0.075	0.776	0.090	0.045	0.014
	(0.031–0.167)	(0.661–0.860)	(0.041–0.185)	(0.015–0.130)	(0.002–0.098)
W	0.050	0.033	0.017	0.783	0.117
	(0.016–0.144)	(0.008–0.124)	(0.002–0.109)	(0.662–0.870)	(0.057–0.225)
G	0	0	0	0	1
	(0–0)	(0–0)	(0–0)	(0–0)	(1–1)
					
Breeding philopatry					
Breeding rookery:					
F	0.996	0.003	0	0.001	0
	(0.988–0.999)	(0.001–0.011)	(0–0)	(0–0.009)	(0–0)
H	0.007	0.989	0.004	0	0
	(0.002–0.026)	(0.966–0.997)	(0.001–0.031)	(0–0)	(0–0)
B	0	0.085	0.855	0	0.060
	(0–0)	(0.012–0.418)	(0.557–0.965)	(0–0)	(0.008–0.333)
W	0.005	0.006	0	0.978	0.011
	(0.001–0.035)	(0.001–0.044)	(0–0)	(0.943–0.992)	(0.003–0.042)
G	0.000	0	0	0.027	0.973
	(0.000–0.000)	(0–0)	(0–0)	(0.009–0.082)	(0.918–0.991)

Five rookeries are listed from south to north, distances (km) between rookeries were F↔H (135), H↔B (105), B↔W (120), and W↔G (75, see Fig 1 for rookery codes and locations). Estimates are from the top model in Table 1C. Rookery fidelity estimates are boxed; unboxed estimates are movement probabilities between rookeries (probabilities of moving *from* areas in rows *to* areas in columns). 95% CI are in parentheses. Natal philopatry and movement probabilities estimate where a female was first observed parous in relation to her natal rookery, breeding philopatry applies to subsequent observations of a female parous in relation to where she was last observed parous.

**Fig 2:**
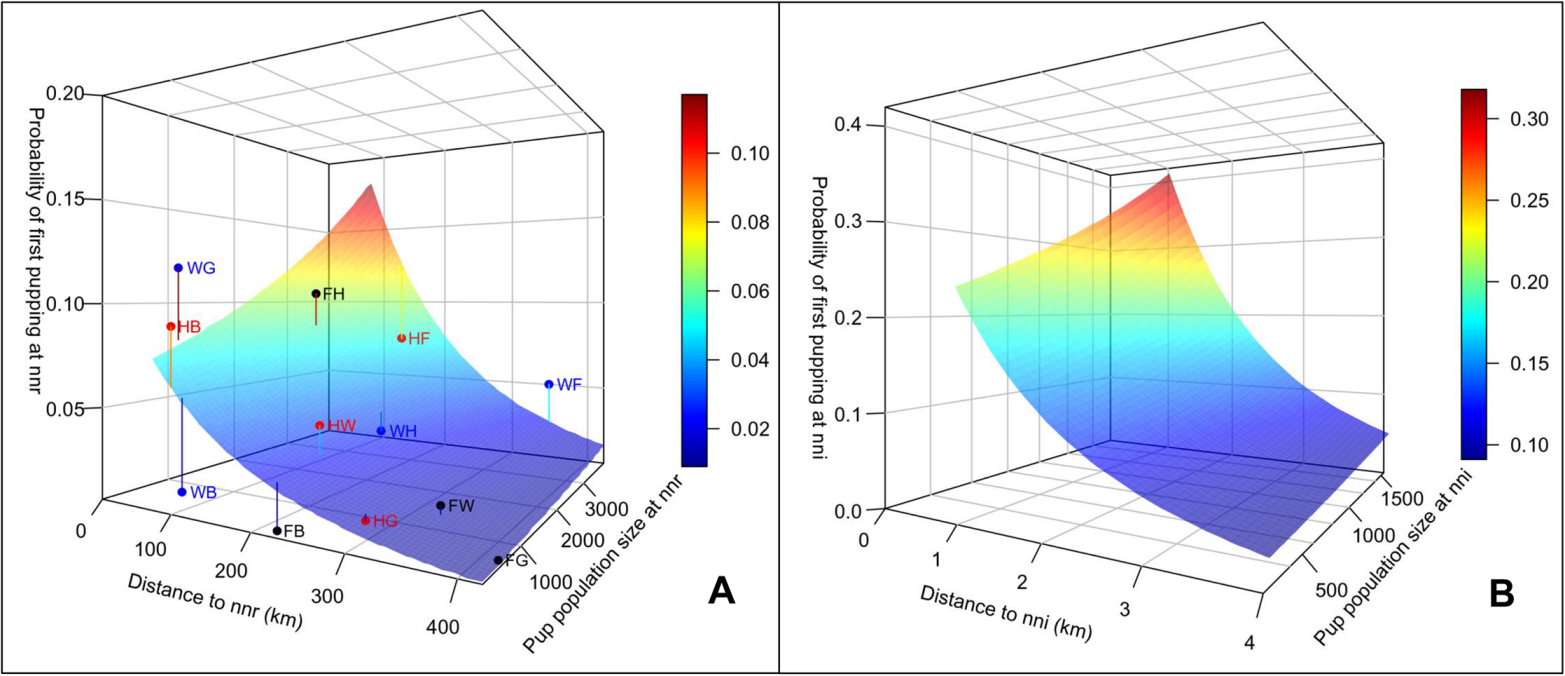
**The probability of Steller sea lion females first pupping at a non-natal rookery (nnr, A) or island (nni, B) by distance from the natal rookery/island and pup population size of the destination rookery/island.** Predicted values were calculated from coefficients estimated in model 39 in Table 1D for A, and in model F9 in Table 1F for B. In A, we show deviations between the predicted values based on population size and distance and the rookery-specific estimates (estimates in Table 2). Deviations are labeled, for example, HF: for movement from natal rookery H to first breeding rookery F, based on rookery codes in the caption for Fig 1.

Because breeding philopatry was nearly 1 for all groups, and natal philopatry was 1 for females born at G, we examined effects of distance and population size on probability of being first observed parous at a non-natal rookery for animals from natal rookeries F, H, and W. The best model with effects of distance and population size on *ψ* was 7x better than the next best model without these effects. The best model included a linear distance effect (note that a linear pattern on the logit scale used in the model is non-linear when transformed to the probability scale, see Fig 2, Table 1D). Population size of the destination rookery was important once distance was included (Table 1D). Females were more likely to move to a less distant than more distant rookery, and to a more than less populous rookery (Fig 2A). Intercepts for F-, H-, and W-born females could be pooled (AICc = 3537.82 for pooled intercept model versus 3541.82 for not pooled) suggesting apparent higher natal philopatry for F than H and W animals (Table 2) may result from more distant and less populous rookeries to move to for F-born females.

### F analysis

*P* varied among the four site groups in the expected manner: in 2014, *p* for 10 year-olds was 0.83 for LN, 0.74 for LS, 0.54 for NR and 0.69 for Islands2 (Table 1E). Reduced boat surveys in 2015 produced very low *p* for boat-surveyed islands in that year: for 10 year-olds, 0.19 and 0.11 at Islands2 and NR, and 0.80 and 0.74 at LN and LS, respectively. Estimates of *S* for the F analysis were nearly identical to those from the SEAK analysis, with all differing by < 0.01.

Natal and breeding philopatry were lower at the smaller spatial scale at F (< 4 km) than at the scale of rookeries (75–420 km). Natal island philopatry was highest at the most populous islands (0.500 and 0.684 at NR and LI) and lower at less populous islands (0.437 and 0.295 at SLR and CHR, Table 3). As in the SEAK analysis, breeding philopatry was higher than natal philopatry, with a similar spatial pattern to natal philopatry: highest at NR and LI (0.926–0.919) and lower at CHR and SLR (0.767–0.858). ER, where no pups were marked and so all marked females breeding were migrants, had the lowest breeding philopatry (0.604). When an even smaller spatial scale (within island) was considered for LI, a sizable portion bred at the non-natal area (LS, 0.295) but a greater number were first observed parous at the natal area than at the non-natal area (LN, 0.389, Table 3). Those that first bred at the non-natal area were more likely to later breed on other islands (0.14) than those that first bred at the natal area (0.04, Table 3). The inclusion of age in *ψ* was also not supported in the F analysis (model F1, Table 1E: AICc = 2837.42 versus same model with age effect: AICc = 2913.38).

**Table 3:** Estimates of natal and breeding philopatry for female Steller sea lions at Forrester Island Complex, 2005 and 2007–15.

			*To*: Breeding island					
*From*:* *	LN		LS	LI	ER	SLR	CHR	NR
Natal philopatry								
Natal island:								
LN	0.389 (0.269–0.524)	0.295		0.684	0.093	0.037	0.056	0.130
		(0.190–0.430)		(0.551–0.795)	(0.039–0.204)	(0.009–0.136)	(0.018–0.159)	(0.063–0.248)
SLR	0.250 (0.097–0.508)	0.125		0.375	0	0.437	0.188	0
		(0.031–0.386)		(0.179–0.623)	(0–0)	(0.225–0.676)	(0.062–0.447)	(0–0)
CHR	0.068 (0.022–0.191)	0.250		0.318	0.114	0.182	0.295	0.091
		(0.144–0.397)		(0.199–0.468)	(0.048–0.245)	(0.094–0.323)	(0.180–0.445)	(0.035–0.218)
NR	0.178 (0.101–0.293)	0.048		0.226	0.097	0.048	0.129	0.500
		(0.016–0.140)		(0.139–0.346)	(0.044–0.199)	(0.016–0.140)	(0.066–0.237)	(0.378–0.622)
Breeding philopatry Breeding island:								
LN	0.888 (0.833–0.926)	0.071 (0.041–0.119)			0.033 (0.013–0.079)	0.002 (0–0.237)	0 (0–0)	0.006 (0.001–0.043)
LS	0.118 (0.073–0.186)	0.740			0.024	0.033	0.085	0
		(0.653–0.812)			(0.007–0.084)	(0.011–0.091)	(0.044–0.156)	(0–0)
LI				0.919	0.029	0.015	0.034	0.003
				(0.881–0.946)	(0.014–0.047)	(0.006–0.039)	(0.018–0.063)	(0.000–0.026)
ER	0.181 (0.092–0.326)	0.032		0.220	0.604	0.072	0.078	0.026
		(0.005–0.178)		(0.119–0.371)	(0.445–0.744)	(0.022–0.217)	(0.024–0.225)	(0.003–0.173)
SLR	0.020 (0.004–0.098)	0.021		0.040	0.018	0.858	0.084	0
		(0.004–0.117)		(0.012–0.125)	(0.003–0.118)	(0.751–0.924)	(0.035–0.189)	(0–0)
CHR	0.023 (0.006–0.083)	0.120		0.136	0.013	0.072	0.767	0.012
		(0.067–0.206)		(0.080–0.218)	(0.002–0.088)	(0.033–0.153)	(0.669–0.843)	(0.002–0.082)
NR	0.010 (0.002–0.056)	0.008		0.018	0.026	0.015	0.015	0.926
		(0.001–0.053)		(0.005–0.062)	(0.008–0.080)	(0.003–0.067)	(0.003–0.068)	(0.866–0.960)

LN = Lowrie Island North, LS = Lowrie Island South, LI = Lowrie Island (LN and LS combined), ER = East Rocks, SLR = Sea Lion Rocks, CHR = Cape Horn Rocks, NR = North Rocks (see Fig 1). Shaded areas indicate movement probabilities for 2 subareas on LI: LN and LS. Estimates are from the top model in Table 1E. Estimates of area fidelity are boxed; unboxed estimates are movement probabilities between areas (probabilities of moving from areas in rows to areas in columns). 95% CI in parentheses.

Although estimated natal philopatry probabilities were lower at the smaller spatial scale of islands than the larger scale of rookeries, a preference for the natal island had statistical support. Generalized linear models suggested philopatry to the natal island was higher than expected after accounting for pup population size at islands. AICc was 174.2 for the model with only pup population size included, and 100.7 for the model with both population size and "natal island" included. Estimates of natal island philopatry (NIP) ranged 1.7–2.7 times that of relative pup abundance (RP) with: 69% (NIP) versus 39% (RP) at LI, 44% versus 16% at SLR, 30% versus 11% at CHR, and 50% and 29% at NR.

For the F analysis, we fit only a linear effect of distance based on results from the SEAK analysis. Similar to the SEAK analysis, the model with effects of distance and population size on movement probabilities was strongly supported (26x better than next model without effects), for both natal and breeding philopatry (Table 1F). As in the SEAK analysis, females were more likely to move to a less distant than more distant rookery, and to a more than less populous rookery (Fig 2B). The intercept could not be pooled across natal islands for either natal or breeding movements (e.g. model F12 versus model F9, Table 1F). Estimated coefficients suggested fewer movements to non-natal islands for LI-born females (*β* = -0.525, 95% CI: -1.240–0.189) than for females born at other islands (CHR: 1.140, 0.309–1.971, SLR: 0.609, -0.503–1.722, NR: 1.032, 0.112–1.952), after accounting for distance and population size effects.

## Discussion

For breeding SSL females born in SEAK, our results suggest moderately high natal philopatry of 0.78–0.86 for most rookeries, consistent with [26], and very high (nearly 1 for most rookeries) breeding rookery philopatry (Table 2). Many SSL juveniles travel widely [26, 28] and juvenile females may encounter multiple suitable locations resulting in reduced natal philopatry. Natal philopatry of 1 was observed for females born at G, in an area with high population growth [48], small dispersal range [Alaska Department of Fish and Game [ADFG], *unpublished data*], high juvenile survival, and larger body size of pups [36], suggesting females may restrict their movements as juveniles and adults in a productive and/or less densely occupied environment. However, the sample size of females born at G was small (*n* = 22 females) and so the high estimate should be verified with a larger sample. Breeding philopatry was particularly low at B (0.855). All females that pupped at B were immigrants from other natal rookeries, and while the result from such a small sample requires more study, it may suggest migrants were more likely to move pupping sites throughout their lifetimes.

The level of movement between natal and breeding sites observed in our study suggests some flexibility in female reproductive strategies that would serve to increase gene flow and improve population resilience [7]. The very high fidelity to pupping sites once females begin to breed suggests familiarity with neighboring females and/or knowledge of the breeding rookery, including the topography of pupping sites and nearby foraging locations, likely improves a female's chance of reproductive success. SSL females with pups have unusually short foraging trip durations compared to other otariids, alternating 0.5 days at sea foraging with 1.5 days attending their pups on land when pups are < 1 month old [37] and therefore reproductive success for this species may be particularly dependent on knowledge of local foraging areas that are very near-shore. Whether higher breeding philopatry results in greater fitness for SSL remains unstudied, but offspring production was higher for females demonstrating greater fidelity to breeding colonies in a Weddell seal population [49].

At the smaller spatial scale of islands within a rookery (F, < 4 km range), estimates of natal philopatry ranged only 0.295–0.685 among natal islands (Table 3). However, a preference for first pupping at the birth island was statistically supported (females were more likely to pup at their natal island than other islands at F if they pupped at F). Similar to rookery-scale estimates, breeding philopatry was higher than natal philopatry at the island-scale, but was less than the ~1.0 estimate observed at the rookery-scale. The least populous island (ER) had lowest breeding philopatry (0.604), and similar to B at the rookery-scale, consisted of only migrants as no pups were marked there. High breeding philopatry was consistent with results from a study of very fine-scale breeding philopatry at a small rookery in the western population (Chiswell Island, Alaska, with up to 80 pups) which found 64% of females gave birth within 5.8 m of their previous pupping site [16].

Female SSL that do not pup at their natal island or rookery, or that change breeding islands among years at F, are more likely to move to more populous and less distant areas. This pattern was present at large (rookeries 75–420 km apart) and small (islands at F 1–4 km apart) spatial scales. The correlation between movement probability and distance is consistent with genetic studies that suggested genetic structure of pups born at different rookeries and geographic distances between rookeries were correlated [21]. The attraction of female SSL to large groups for reproduction may suggest a positive correlation between quality (whether in terms of pupping sites, mate choice, and nearby foraging grounds) and rookery size, or that animal density may improve female fitness, or the relationship simply results from the behavioral tendency of SSL to be attracted to conspecifics. More study is required to address causes underlying this pattern, but a positive fitness effect due to higher animal density was suggested by pup survival at F being highest after the time of maximum non-pup counts, possibly due to higher density limiting pup movement which prevented pups from being washed into surf and drowning [47]. If superior males occur at dense sites, females' fitness may also benefit. A positive correlation between distance to foraging grounds (quality) and density at the small spatial scale may also be suggested by the most populous islands being in the north at F, and, for nine adult females with pups captured and instrumented with satellite dive recorders at four of the five islands at F, most foraging trips during the breeding season were oriented to the north and northeast [50]. Regardless of the causes, the consequences of these patterns include correlation of population dynamics at multiple spatial scales, the tendency towards large refugia, the slowing of population growth, and a resulting distribution in which SSL occupy only a portion of the suitable breeding habitat available to them [7].

Our study addresses fidelity to pupping sites, rather than mating sites. SSL females mate on average 11–12 days after parturition [39, 51], and so females with pups mate at their pupping rookery. No branded females with pups were observed before 1 Aug at > one rookery with their pup in the same breeding season. But, as reproductive rates are 0.63–0.70 for this species [34, 52–53], females may not give birth every year. After summarizing reproductive status each year as the most definitive behavior observed during June–July for the 369 females included in our study, there were 128 female *x* year sightings with only a dependent juvenile observed with the female, suggesting in these cases, that the pup may have been missed in all surveys, that the female may not have given birth that year, or that the females' pup may have died soon after birth and she reunited with her previous-years' offspring. In SSL, offspring often wean at ~age 1, but some may not wean until 2–3 years of age, and few females may nurse both a new pup and a juvenile simultaneously for a period during the breeding season [34]. Of these 128, 39% were seen at their preferred pupping rookery with the juvenile, 20% were seen at another rookery with the juvenile, and 41% were seen at haul-outs with the juvenile [ADFG *unpublished data*]. Therefore, females without pups may move more (within and between years) than females with pups and may mate at other rookeries and haul-outs rather than their preferred pupping rookery. Movement estimates based only on females with pups therefore likely underestimate the amount of female-mediated gene-flow that occurs in SEAK, including the contribution of matings at haul-outs and other rookeries in years when females do not give birth.

SSL share much of their range with the historical range of northern fur seals (*Callorhinus ursinus*) [54]. Similar to northern fur seals which experienced catastrophic population decline following over-hunting which resulted in range collapse (reviewed by [55]), haplotype diversity was high in Steller sea lions with no signs of genetic bottlenecks [21], despite severe population decline through much of its range [29, 30]. The genetic data suggest that relatively warm periods have promoted population expansion and dispersal of SSL by increasing available rookery habitat [56]. We expect the dispersal capabilities of breeding SSL females, such as those measured by this study, will assist population recovery. In fact, the longest dispersal distances of females pupping at non-natal rookeries have been observed for females from Sugarloaf Island in the recovering western population, traveling up to 1030 km to W to pup [28]. The range collapse of northern fur seals in the North Pacific was not accompanied by loss of genetic diversity likely because of the persistence of an abundant population at a refugium and the species capabilities for high dispersal rates [55]. Behavioral plasticity in life-history strategies of northern fur seal females, particularly flexibility in the length of the weaning period to allow longer offspring rearing periods to buffer against lower productivity and interannual variability, allowed northern fur seals to exploit a greater geographic area including temperate to sub-arctic conditions, but this plasticity appears to have been lost with range collapse [55, 57]. SSL also demonstrate plasticity in dispersal rates ([26, 28], this study) and the length of the offspring rearing period (1–3+ years) [34] and links between these processes and resilience of SSL populations deserves further study.
