# Correction: Natal and breeding philopatry of female Steller sea lions in southeastern Alaska

**DOI:** 10.1371/journal.pone.0196412

**Published:** 2018-04-20

**Authors:** Kelly K. Hastings, Lauri A. Jemison, Grey W. Pendleton, Kimberly L. Raum-Suryan, Kenneth W. Pitcher

In the F analysis subsection of the Results section, the first and second sentences incorrectly refer to Islands2. They should instead refer to Islands1. The correct sentences are: *P* varied among the four site groups in the expected manner: in 2014, *p* for 10 year-olds was 0.83 for LN, 0.74 for LS, 0.54 for NR and 0.69 for Islands1 (Table 1E). Reduced boat surveys in 2015 produced very low *p* for boat-surveyed islands in that year: for 10 year-olds, 0.19 and 0.11 at Islands1 and NR, and 0.80 and 0.74 at LN and LS, respectively.

There is an error in the first sentence of the third paragraph of the Discussion section. The correct sentence is: At the smaller spatial scale of islands within a rookery (F, < 4 km range), estimates of natal philopatry ranged only 0.295–0.684 among natal islands (Table 3).

In Table 2, the boxes around the rookery fidelity estimates are difficult to see. Please see a corrected Table 2 here.

**Table 2:** Estimates of natal and breeding philopatry for female steller sea lions in southeastern Alaska, 2002–15.

	*To*: Breeding rookery (South → North)				
*From*:	F	H	B	W	G
**Natal philopatry** **Natal rookery:**					
**F**	0.859	0.105	0.009	0.018	0.009
	(0.807–0.899)	(0.070–0.152)	(0.002–0.036)	(0.007–0.047)	(0.002–0.036)
**H**	0.075	0.776	0.090	0.045	0.014
	(0.031–0.167)	(0.661–0.860)	(0.041–0.185)	(0.015–0.130)	(0.002–0.098)
**W**	0.050	0.033	0.017	0.783	0.117
	(0.016–0.144)	(0.008–0.124)	(0.002–0.109)	(0.662–0.870)	(0.057–0.225)
**G**	0	0	0	0	1
	(0–0)	(0–0)	(0–0)	(0–0)	(1–1)
**Breeding philopatry** **Breeding rookery:**					
**F**	0.996	0.003	0	0.001	0
	(0.988–0.999)	(0.001–0.011)	(0–0)	(0–0.009)	(0–0)
**H**	0.007	0.989	0.004	0	0
	(0.002–0.026)	(0.966–0.997)	(0.001–0.031)	(0–0)	(0–0)
**B**	0	0.085	0.855	0	0.060
	(0–0)	(0.012–0.418)	(0.557–0.965)	(0–0)	(0.008–0.333)
**W**	0.005	0.006	0	0.978	0.011
	(0.001–0.035)	(0.001–0.044)	(0–0)	(0.943–0.992)	(0.003–0.042)
**G**	0.000	0	0	0.027	0.973
	(0.000–0.000)	(0–0)	(0–0)	(0.009–0.082)	(0.918–0.991)

Five rookeries are listed from south to north, distances (km) between rookeries were F↔H (135), H↔B (105), B↔W (120), and W↔G (75, see Fig 1 for rookery codes and locations). Estimates are from the top model in Table 1C. Rookery fidelity estimates are boxed; unboxed estimates are movement probabilities between rookeries (probabilities of moving *from* areas in rows *to* areas in columns). 95% CI are in parentheses. Natal philopatry and movement probabilities estimate where a female was first observed parous in relation to her natal rookery, breeding philopatry applies to subsequent observations of a female parous in relation to where she was last observed parous.
